# Laminins Regulate Placentation and Pre-eclampsia: Focus on Trophoblasts and Endothelial Cells

**DOI:** 10.3389/fcell.2020.00754

**Published:** 2020-08-07

**Authors:** Min Liu, Yangxue Yin, Hongbiao Yu, Rong Zhou

**Affiliations:** Department of Obstetrics and Gynecology, West China Second University Hospital, Sichuan University; Key Laboratory of Birth Defects and Related Diseases of Women and Children, Sichuan University, Ministry of Education, Chengdu, China

**Keywords:** pre-eclampsia, laminin, trophoblast, endothelial dysfunction, placenta

## Abstract

Pre-eclampsia is a systemic vascular disease characterized by new-onset hypertension and/or proteinuria at ≥20 weeks of gestation and leads to high rates of maternal and perinatal morbidity and mortality. Despite the incomplete understanding of pre-eclampsia pathophysiology, it is accepted that insufficient spiral artery remodeling and endothelial dysfunction are major contributors. Laminins (LNs) are a vital family of extracellular matrix (ECM) molecules present in basement membranes that provide unique spatial and molecular information to regulate implantation and placentation. LNs interact with cell surface receptors to trigger intracellular signals that affect cellular behavior. This mini-review summarizes the role of LNs in placental development during normal pregnancy. Moreover, it describes how LN deficiency can lead to the pre-eclampsia, which is associated with trophoblast and vascular endothelial dysfunction. New research directions and the prospect of clinical diagnosis of LN deficiency are discussed, and the gaps in basic and clinical research in this field are highlighted.

## Introduction

Pre-eclampsia, is a pregnancy disease mainly characterized by gestational hypertension (≥140/90 mmHg) at gestation ≥20 weeks (Homer et al., 2008); it affects 3–5% of pregnancies and is more common in low- and middle-income countries (Ananth et al., 2013; Mol et al., 2016). Once diagnosed, the only effective treatment is cesarean delivery. Even if other appropriate treatment is administered for in pregnancy hypertensive diseases, there are still long-term effect on the children and mothers (Mate et al., 2019). Pre-eclampsia is a placental disease with two stages. The first is placental structure abnormality characterized by insufficient spiral artery remodeling and reduced placental perfusion. During a normal pregnancy, placentation begins with the recognition and adhesion of the trophectoderm to the uterine epithelium; trophoblast stem cells originate from the embryonic trophectoderm and differentiate into various trophoblast lineages [as reviewed in Silva and Serakides (2016)]. A recent review (Aplin et al., 2020) clearly summarized the steps in normal and failed uterine spiral arterial conversion. Cytotrophoblast cells invade into the decidua (interstitial trophoblast), and then the wall of maternal uterine spiral artery is destroyed, including the endothelium and smooth muscle cell. Extracellular matrix (ECM) components of the vessel wall are replaced with a fibro-fibrinoid structure, within which residual endovascular trophoblast often remains embedded. The remodeled uterine spiral arteries are characterized by lower resistance and higher flow, and they perfuse the placental villi to support placental and fetal development (Brosens et al., 2019). However, conversion failure results in poor blood supply to the intervillous spaces (Brosens et al., 2011). The second stage is manifestation of maternal hypertension and proteinuria with systemic endothelial dysfunction. An imbalance between pro- and anti-angiogenic proteins can lead to increased blood pressure and pre-eclampsia in pregnancy. Some vascular endothelial protective factors [e.g., nitric oxide/nitric oxide synthase, vascular endothelial growth factor (VEGF)] are key factors in maintaining blood pressure, vascular endothelial environmental stability, and angiogenesis (Cudmore et al., 2006). Conversely, vascular endothelial damage factors [e.g., soluble fms-like tyrosine kinase 1 (sFlt-1), soluble endoglin (sEng)] exert anti-angiogenetic effects (De Falco, 2012; Graupner et al., 2019). The transition between the two stages is thought to be due to the release of such factors from the abnormally developed placenta into maternal circulation.

Normal placentation depends on cell-to-cell and cell-to-ECM interactions. The ECM consists of several unmodified and conjugated proteins such as collagen, fibronectin, laminins (LNs) and others that provide a microenvironment for placental cells and regulate cell functions including proliferation, migration, invasion, and signaling (Kim et al., 2014; Graubner et al., 2018). LNs are ECM molecules that comprise a family of glycoproteins found predominantly in basement membranes; they interact with cell surface receptors that transmit intracellular signals that regulate cellular behavior (Lala et al., 2012). Some studies have shown that LN deficiency is associated with many human diseases (McGowan and Marinkovich, 2000). Animal and human studies have shown that LNs are involved in placental development and affect the function of cytotrophoblastic cells (Kuo et al., 2018). This review outlines the critical role of LNs in placental development and molecular mechanisms involved in pre-eclampsia development.

## Structure and Function of Laminin

**FIGURE 1:**
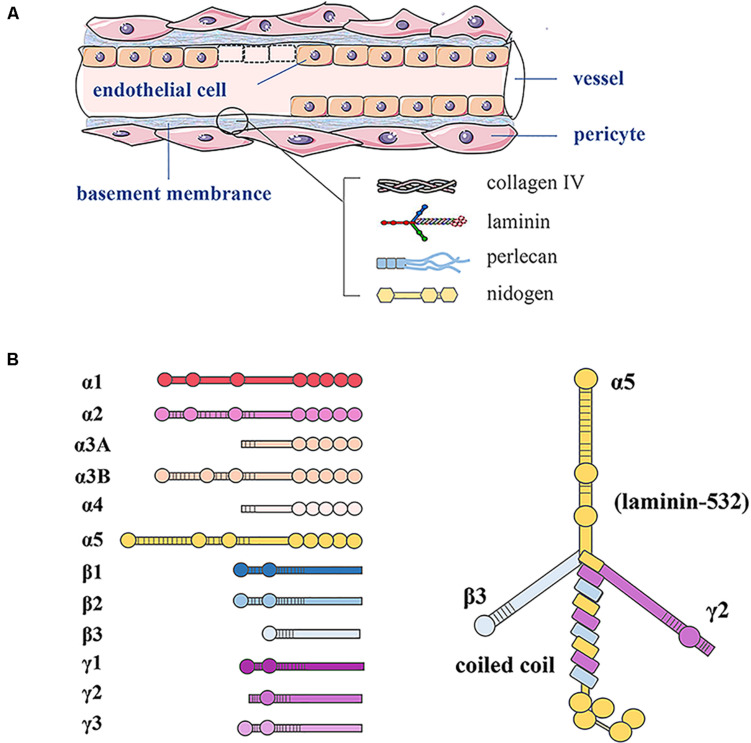
Basement membrane constituents in vessels and LN structure. **(A)** In blood vessels, the basement membrane separates endothelial cells from supporting cells such as pericytes and connective tissue. The basement membrane consists of LN, type IV collagen, perlecan, and nidogen. **(B)** LN as a trimeric protein containing five α, three β, and three γ chains; all laminin chains share a common domain structure with a number of globular and rod-like domains.

As shown in Figure 1, the basement membrane is the thin sheet of ECM that underlies epithelial and endothelial cells and surrounds muscle cells, Schwann cells, and connective tissue. LNs are ECM molecules that comprise a family of glycoproteins found predominantly in basement membranes, as a trimeric protein containing five α, three β, and three γ chains that are genetically distinct (Domogatskaya et al., 2012). The trimers are named according to their composition of α, β, and γ chains. For example, LN-523 is made of α5, β2, and γ3 chains (Tzu and Marinkovich, 2008).

Laminins influence cell adhesion, migration, phenotype maintenance, survival, and differentiation (Durbeej, 2010). The LNβ3 chain mediates apoptosis, proliferation, invasion, and metastasis of pancreatic cancer cells *via* the phosphoinositide 3-kinase (PI3K)/Akt signaling pathway (Zhang et al., 2019). Difficulties in isolating LN isoforms from tissues have hampered studies of their biological roles, and most information has come from research on diseases caused by gene mutations or using knockout mice. For instance, LNα5 gene mutation leads to defects in neural tube closure, finger separation, placentation, kidney formation, pulmonary lobe separation, hair morphogenesis, and intestinal smooth muscle differentiation (Miner and Li, 2000; Nguyen et al., 2002; Bolcato-Bellemin et al., 2003). Integrins, dystroglycan, syndecans, and Lutheran/basal cell adhesion molecule (BCAM) are cellular receptors for LNs (Desgrosellier and Cheresh, 2010; Aumailley, 2018; Jin et al., 2019). The biological effects of LNs are presumably largely mediated by surface receptors that link LN matrices to intracellular signaling pathways [as reviewed in Durbeej (2010)]. One group suggested that LNs promote the differentiation and proliferation of rat embryonic stem cells into cardiomyocytes by interacting with the integrin pathway (Wang et al., 2019). Lutheran/BCAM is oncogenic in human urothelial cancers in the presence of its ligands LN-511, LN-521 (Chang et al., 2017).

## Laminins and Placentation

Laminin is the basement membrane component that initially promotes cell adhesion and angiogenesis in early embryonic development (Givant-Horwitz et al., 2004). LN-111 affects cell-cell adhesion by changing the localization of vascular endothelial cadherin (Miner et al., 2004). LN activity is closely correlated with the blastocyst implantation window. LN levels in the basement membrane of the placental villi are decreased in late pregnancy compared to early pregnancy, due to the completion of the adhesion and infiltration process of placental trophoblasts (Turpeenniemi-Hujanen et al., 1992). Trophectoderm defects can lead to improper embryo implantation, and abnormal placental formation has deleterious effects on later stages of pregnancy, potentially causing conditions such as pre-eclampsia and intrauterine growth restriction (Red-Horse et al., 2004). Thus, LN is essential for proper embryo implantation and subsequent placental formation. One study showed that cytotrophoblast invasion was evident in some LNβ1^–/–^ mouse embryos, but the extent of invasion was greatly reduced compared with normal embryos (Miner et al., 2004). Moreover, cytotrophoblast behavior and differentiation may be affected by interactions with LN-specific isoforms, thereby regulating the structural formation of the placenta, and attachment of invasive trophoblasts to the basement membrane can trigger the expression of matrix metalloproteinases (MMPs) necessary for their separation from the basement membrane, degradation, and migration (Ramos et al., 1990). LN knockout (LNα1, LNβ1, LNβ5) is embryonic lethal in mice. During placental development, LNα5 knockout mice exhibit malformed placental labyrinths and abnormal trophoblast differentiation (Miner et al., 1998; Sekiguchi and Yamada, 2018). In addition, Pijnenborg et al. (1996) described a contribution of LNs to fibrinoid deposition within physiologically remodeled spiral arteries in baboons. Collectively, these findings indicate that LN is particularly important for the subsequent differentiation of cytotrophoblast cells and spiral artery remodeling.

Laminin activates cellular surface receptors to assist in its biological functions, and most recent studies have focused on integrins and LN-111 receptor (LR1). LNs and integrins differ in their binding specificities and affinities (typically including integrins α3β1, α6β1, α7β1, and α6β4), and integrin expression patterns are strictly regulated during placental development (Maltepe and Fisher, 2015). For example, integrins α6β1 and α7β1 bind to LNs and are abundant in the basement membrane of the endometrial epithelium during the implantation window (Merviel et al., 2001). Integrin serves as a master switch for cytotrophoblast transition from a proliferative (α6β4 and α5β1) to an invasive (α1β1) phenotype; this provides a foundation for studying pathological conditions characterized by insufficient trophoblast invasion (Kilburn et al., 2000). Increased LR1 expression in decidual cells of partial and complete moles may enable trophoblasts or other cells (e.g., endothelial cells and lymphocytes) to control invasion by modifying the structure of its primary ligand, LN-111 (Nelson et al., 2008; Kurdoglu et al., 2009).

## Laminins and Pre-Eclampsia

Trophoblast invasion of the uterine decidua and maternal vascular system is the key to successful embryo implantation and pregnancy, and LNs play a critical role in this multistep process. Despite an incomplete understanding of pre-eclampsia pathophysiology, it is generally though that the primary mechanisms are insufficient spiral artery remodeling and endothelial dysfunction (Gauster et al., 2009). Thus, we mainly analyze the relationship between LNs and pre-eclampsia from the perspective of improper regulation of LNs on biological behaviors of trophoblasts and vascular endothelial function.

### Abnormal Biological Functions of Trophoblasts

In recent years, abnormal trophoblast function has been proposed to play a major role in pre-eclampsia pathogenesis. Potential cause of trophoblast invasion defect in pre-eclampsia including abnormal levels of cytokines, growth factors, placental factors, and interleukins (Moser et al., 2018). The ratio of type IV collagen to LNs is significantly higher in pre-eclamptic placentas compared to controls, most likely reflecting a lower level of LNs in the villi (Risteli et al., 1984). A study of microarray datasets reported that decreased expression of the LNα2 chain in the basal plate may be involved in the development of pre-eclampsia (Winn et al., 2011). Proteomic analysis also showed that significantly lower LN levels in placental trophoblastic cells from pre-eclampsia pregnant women compared to controls, including LNβ2 (12.5-fold), LNα5 (9.1-fold), and LNγ1 chain (5.6-fold) (Ma et al., 2014). Pre-eclampsia placentas exhibit lower LNα5 chain levels, and this deficiency inhibits proliferation and invasion but promotes cell apoptosis in HTR-8/SVneo cells, possibly through inhibition of the PI3K/AKT/mammalian target of rapamycin (mTOR) signaling (Zhang et al., 2018). Downregulation of LNα4 also inhibited the proliferation, migration, and invasion of trophoblasts to suppress the expression of vascular factors in HTR-8/SVneo cells (Ji et al., 2019). Moreover, Shan et al. reported significantly lower expression of LNα4 in human pre-eclampsia placentas compared to control, and LNα4 plays a crucial role in trophoblast differentiation and invasion (Shan et al., 2015). Such changes in placental LN levels could weaken trophoblast attachment to the underlying basement membrane and also modify villi permeability and exchange properties.

Signal transduction *via* LRs in normal and malignant trophoblasts influence their adhesion to the basement membrane (Burrows et al., 1995). LR1 is a non-integrin-type receptor for LN-111 that exerts many effects on cell migration, adhesion, differentiation, invasion, signaling, and tumor metastasis. Decreased immunohistochemical staining for LR1 protein in cytotrophoblasts and syncytiotrophoblasts was observed in pre-eclamptic placentas, and decreased LR1 in cytotrophoblasts might lead to shallow trophoblastic invasion (Kurdoglu et al., 2011). LR1 knockdown remarkably suppressed the migration and invasion potential of JEG3 cells by reducing MMP-2 and MMP-9 activities, suggesting a possible pathogenetic mechanism of pre-eclampsia (Wang et al., 2013, 2015). Zhou et al. (1997) reported that trophoblastic invasion of the uterine spiral artery is limited because there is no decrease in integrin α6β4 and no increase in integrin α1β1 in pre-eclampsia pregnancies. Another group observed that integrin β1 mRNA was decreased in placental tissue from pre-eclampsia subjects, and then verified its involvement in dysregulating trophoblast cell invasion, survival, and angiogenesis in HTR8/Svneo cells (Li et al., 2013). The slightly decreased integrins αv and β1 subunits in the cytotrophoblasts may be a structural basis for poor placental perfusion in pre-eclampsia (Vatansever et al., 2003). Collectively, these findings demonstrate that integrin expression pattern are highly regulated during cytotrophoblast differentiation, and alterations in this process are the pathological basis of endovascular trophoblast invasion into the uterine spiral arteries.

### Vascular Endothelial Dysfunction

Maternal endothelial dysfunction caused by placental factors (pro- and anti-angiogenic proteins) has long been considered one of the pathophysiological factors underlying severe pre-eclampsia (Roberts and Hubel, 2009). However, whether angiomodulatory imbalance is a consequence of an underlying pathology that is common to several different types of placental dysfunction is still unclear, as is whether it reflects a specific abnormality of earlier placental development (Aplin et al., 2020). There is now sufficient evidence to suggest that tissue-specific of LNs and their associated signaling regulates cell behavior and angiogenesis [as reviewed in Simon-Assmann et al. (2011)]. Native LN-111 from the human placenta can promote the formation of interconnected vascular networks among human umbilical vein endothelial cells (HUVECs) (Hackethal et al., 2018). LNα4 chain was strongly expressed in endothelial cells in placental villi and decidua, and its expression was decreased in third-trimester pre-eclamptic placentas (Thyboll et al., 2002). Moreover, LNα4 small-interfering RNA transfection and hypoxia-reoxygenation intervention reduced HUVEC migration and tube formation abilities by activating mitogen-activated kinase signaling (Shan et al., 2015). Down-regulated LNα5 is observed in pre-eclampsia placentas and inhibits HUVECs proliferation, migration, and angiogenesis through PI3K/AKT/mTOR pathways (Zhang et al., 2020). However, there are no data to indicate that the endothelial-derived LNα5 chain is involved in initiating the process as its expression occurs in the later stages of angiogenesis (Langen et al., 2017) when it plays critical roles in vascular stability, maturation, and barrier function (Sorokin et al., 1997; Liu et al., 2018). The above findings indicate that LNs are critical factors for endothelial cells to undergo angiogenesis and differentiate into interconnected tubules. LN deficiency may directly lead to abnormal angiogenesis and pathologic placental development. LNs also regulate angiogenesis through different vascular factors. Glomerular endothelial lesions associated with pre-eclampsia are caused by the blocking VEGF/VEGF receptor (VEGFR) and transforming growth factor-β/endoglin signaling pathways by sFlt-1 and sEng, respectively, leading to glomerular endothelial pore loss, cell swelling, and proteinuria (Abrass et al., 2006). A lack of LNα3 in mice or LNβ3 in humans leads to similar defects (Hata et al., 2005). Downregulation of αvβ3 integrin reduces the priming of endothelial cells and tubulogenesis by inhibiting VEGFR activation (Helal-Neto et al., 2016). LN-111 induces expression of pro-angiogenic molecules, such as VEGF and Cxc chemokine receptor 4, thus leading to increased angiogenesis and tumor growth (Mammadova-Bach et al., 2018). These lines of evidence suggest that LNs can directly and indirectly cause vascular endothelial dysfunction and induce severe pre-eclampsia.

The effect of LR signaling on endothelial cell functions is also important. Interaction between α6β1 integrin and LN-411 might promote endothelial cell migration *in vivo*, since their expression patterns overlap with those of newly formed capillaries, where endothelial cells are actively migrating (Sixt et al., 2001). Hypoxia transcriptionally upregulates angiogenic integrins (αv, β1, β3, and β5) in microvascular endothelial cells along with promoting migration and tube formation in human microvascular endothelial cells (Befani and Liakos, 2017). However, the relationship between LR-induced endothelial dysfunction and pre-eclampsia has not been investigated in detail.

## Future Perspectives

To date, most LN signaling studies in pre-eclampsia have focused on trophoblast function, but a few have investigated other aspects. One found that miR-126-3p promoted matrix-dependent perivascular cell attachment, migration, and intercellular interaction (Pitzler et al., 2016). LN-111 is capable of inducing epigenetic changes by inhibiting Dnmt1 expression and preventing methylation of the E-cadherin promoter, ultimately promoting metastatic colonization during breast cancer (Benton et al., 2009). However, there is no clear epigenetic mechanism by which LNs regulate occurrence of pre-eclampsia pathogenesis. It is also noteworthy that vascular endothelial cells express only two LN isoforms: LN-411 and LN-511. LN-411 is expressed by all endothelial cells regardless of their developmental stage, and its expression is strongly upregulated by cytokines and growth factors involved in inflammatory events. LN-511 is primarily detectable in endothelial cell basement membranes of capillaries and venules beginning 3–4 weeks postnatal, and its expression is only upregulated by strong proinflammatory signals (Hallmann et al., 2005). Moreover, the ECM can regulate chemokine production in damaged endothelial cells (Zimmerlin et al., 2013). Thus, we hypothesized that LNs expressed by endothelial cells might be involved in the inflammatory response during pre-eclampsia pathogenesis.

Mortality from pre-eclampsia has decreased significantly in the United States due to increased prenatal monitoring and early intervention (Ghossein-Doha et al., 2018). Therefore, it is of considerable significance to explore and identify biomarkers for the detection or control of pre-eclampsia and to develop targeted strategies. Serum or plasma angiogenic factors and cytokines appear to be a reliable risk-stratification approach among women with suspected pre-eclampsia, such as sFlt-1, sEng, placental growth factor (PlGF), interleukin-6, and the sFlt-1/PlGF ratio (Chaiworapongsa et al., 2014). Serum LN level is an important index to assess the degree of liver fibrosis in chronic hepatitis (Lebensztejn et al., 2004). So, could LN be a promising biomarker for diagnosing pre-eclampsia? There is a paucity of prospective studies in this filed with both large sample sizes and well-defined, strict inclusion and exclusion criteria. Furthermore, it is necessary to develop specific and sensitive assays for clinical measurement of LNs.

## Conclusion

**FIGURE 2:**
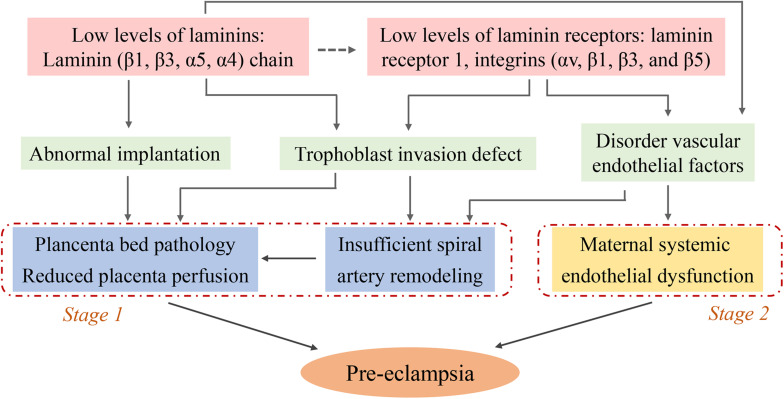
Schematic diagram illustrating the potential causes for the low level of laminins induced- pre-eclampsia: focus on trophoblasts and endothelial cells. The decreased levels of serum LN and placental LN expression (pale pink) contribute to abnormal implantation, trophoblast invasion defect, and disorder vascular endothelial factors (pale green). These pathophysiological states lead to placenta bed pathology, insufficient uterine spiral artery remodeling (blue, known as stage 1 of pre-eclampsia) and maternal systemic endothelial dysfunction (yellow, known as stage 2 of pre-eclampsia). The figure leaves open the possibility that some LNs might play the role by binding to their receptors (dotted arrow, pale pink), while the role of these receptors is clearly known (solid arrow).

Laminins play a critical role in pre-eclampsia as demonstrated by a series of clinical and basic studies. Serum LN levels and placental LN expression are decreased in pre-eclamptic pregnancies. Low LN levels are able to induce abnormal biological functions of placental trophoblast cells and vascular endothelial dysfunction, which lead to implantation failure, inappropriate placental spiral artery remodeling and placental vascular injury, these are the main pathological bases for pre-eclampsia. Moreover LRs, especially integrin-type receptor-mediated signaling pathways may be important contributors (summarized in Figure 2). However, the specific molecular mechanisms are complex and unclear, and a more detailed understanding of the signaling mechanisms is needed. This review proposed a possible mechanism of altered LN-induced pre-eclampsia, including epigenetic modifications, cytokines, and inflammatory events. Finally, LNs are promising diagnostic and prognostic biomarkers for this disease. Additional studies can help guide the use of LN clinical diagnosis and identify prevention and treatment targets for pre-eclampsia.

## Author Contributions

ML and RZ designed the outline of the review and wrote the initial manuscript. All authors edited and approved the final manuscript.

## Conflict of Interest

The authors declare that the research was conducted in the absence of any commercial or financial relationships that could be construed as a potential conflict of interest.
